# Fast whole-slide cartography in colon cancer histology using superpixels and CNN classification

**DOI:** 10.1117/1.JMI.9.2.027501

**Published:** 2022-03-14

**Authors:** Frauke Wilm, Michaela Benz, Volker Bruns, Serop Baghdadlian, Jakob Dexl, David Hartmann, Petr Kuritcyn, Martin Weidenfeller, Thomas Wittenberg, Susanne Merkel, Arndt Hartmann, Markus Eckstein, Carol Immanuel Geppert

**Affiliations:** a Fraunhofer Institute for Integrated Circuits IIS, Image Processing and Medical Engineering Department, Erlangen, Germany; b Friedrich-Alexander-University, Erlangen-Nuremberg, Department of Computer Science, Erlangen, Germany; c University Hospital Erlangen, Department of Surgery, FAU Erlangen-Nuremberg, Erlangen, Germany; d University Hospital Erlangen, Comprehensive Cancer Center Erlangen-EMN (CCC), FAU Erlangen-Nuremberg, Erlangen, Germany; e University Hospital Erlangen, Institute of Pathology, FAU Erlangen-Nuremberg, Erlangen, Germany

**Keywords:** deep learning, colorectal cancer, histological tissue segmentation, superpixel segmentation

## Abstract

**Purpose:**

Automatic outlining of different tissue types in digitized histological specimen provides a basis for follow-up analyses and can potentially guide subsequent medical decisions. The immense size of whole-slide-images (WSIs), however, poses a challenge in terms of computation time. In this regard, the analysis of nonoverlapping patches outperforms pixelwise segmentation approaches but still leaves room for optimization. Furthermore, the division into patches, regardless of the biological structures they contain, is a drawback due to the loss of local dependencies.

**Approach:**

We propose to subdivide the WSI into coherent regions prior to classification by grouping visually similar adjacent pixels into superpixels. Afterward, only a random subset of patches per superpixel is classified and patch labels are combined into a superpixel label. We propose a metric for identifying superpixels with an uncertain classification and evaluate two medical applications, namely tumor area and invasive margin estimation and tumor composition analysis.

**Results:**

The algorithm has been developed on 159 hand-annotated WSIs of colon resections and its performance is compared with an analysis without prior segmentation. The algorithm shows an average speed-up of 41% and an increase in accuracy from 93.8% to 95.7%. By assigning a rejection label to uncertain superpixels, we further increase the accuracy by 0.4%. While tumor area estimation shows high concordance to the annotated area, the analysis of tumor composition highlights limitations of our approach.

**Conclusion:**

By combining superpixel segmentation and patch classification, we designed a fast and accurate framework for whole-slide cartography that is AI-model agnostic and provides the basis for various medical endpoints.

## 1 Introduction

With the introduction of slide scanning systems into pathological workflows, the prerequisite has been met to introduce machine learning algorithms into diagnostic routines. Due to their large size of over 10 billion pixels, however, digitized histopathological whole-slide-images (WSIs) pose a challenge to automatic image analysis approaches. When working with such large images, technicians are oftentimes confronted with compromising computational efficiency for segmentation and classification accuracy. Especially in the clinical environment, however, both sides of the coin are equally desirable. This work focuses on how semantic segmentation of tissue classes can be executed efficiently. We present an algorithm for the analysis of large-scale microscopic images which utilizes local pixel dependencies to achieve high classification accuracy, while maintaining reasonable computational complexity. We propose to introduce clustering into superpixels prior to classification which helps to model underlying biological structures. Furthermore, we present a technique of inferring superpixel classification labels using neural network classification. Using supervised learning and a hand-annotated database of 159 slides of colon resection specimens stained with hematoxylin and eosin (H&E) dye, our solution is trained to distinguish seven tissue classes. The multiclass analysis of tissue facilitates a further evaluation of tumor composition and growth progression such as deriving the invasion front, which we only touch upon in this work, but do not cover in depth.

Beyond the general research question of how whole-slide cartography can be performed efficiently, this work aims to answer the following more concrete questions. Can superpixel clustering prior to patch-based classification be utilized to achieve a speed-up? How large is the speed-up compared with sole patch-based analysis and what is the impact on the segmentation accuracy? Does this approach work equally well for all tissue classes? Is it necessary and beneficial to classify all patches inside a superpixel or is it sufficient to classify only a subset? If so, what is the impact on the speed-up and accuracy and where is a good balance point? Considering medical end points, can the generated tissue map already be used to derive the tumor invasive margin? How accurately can the tumor area be calculated? Is the tumor composition (necrosis, active tumor cells, tumor stroma, and mucus) accurately differentiated?

## 2 Related Work

In the following paper, an overview of recent work in the field of semantic image segmentation and applications to pathological image data is provided. Furthermore, technically related approaches that combine superpixel clustering and subsequent classifications are briefly elaborated.

### 2.1 Semantic Segmentation

Semantic image segmentation describes the process of inferring pixelwise classification labels to generate a two-dimensional (2D) classification output. Due to their large size, WSIs are always divided into smaller image patches which are analyzed individually. In general, two approaches for the semantic segmentation of WSIs can be distinguished: each image patch can be analyzed by a classification or segmentation network. The former predicts a single class-label for the whole image patch and after reassembling the classified patches, a segmentation mask of the WSI can be obtained. This classification-based approach has been applied both in a nonoverlapping manner,^1^^,^^2^ creating coarse segmentation masks, and, at the cost of higher computation times, in a sliding-window manner as neighborhood around each image pixel.^3^ To incorporate image information on various scales, multiple resolutions can be integrated into a classification-based analysis.^4^^–^^6^

For the latter approach, based on the segmentation of image patches, special fully convolutional neural network^7^ architectures such as U-Net^8^ or SegNet^9^ are typically used. These architectures employ encoder–decoder structures for the prediction of 2D segmentation outputs and have been used for scene^9^ and biomedical image segmentation.^8^^,^^10^^–^^12^ Encoder–decoder-based approaches are able to generate a segmentation output with a high granularity that can only be achieved by classification-based approaches when classifying each image pixel with its neighborhood as individual patches. However, these approaches entail high computational complexity and require extensive hardware resources. Oskal et al.,^8^ for instance, reported inference times of up to 18 min per WSI when using an NVIDIA Tesla P100 GPU and Khened et al.^11^ 30 to 75 min per WSI with an NVIDIA Titan-V GPU. These complex hardware requirements might not be attainable in a clinical setting and faster computation times are often desired.

### 2.2 Applications in Digital Pathology

In the field of digital pathology, machine learning algorithms have increasingly gained importance for answering pathological research questions. Bychkov et al.,^13^ for instance, proposed a convolutional neural network (CNN)-based approach for directly predicting 5-year disease-specific survival for patients with colorectal cancer merely from tissue microarray cores.

For the semantic segmentation of WSIs, two standard approaches can be distinguished: cell-based and texture-based methods. Sirinukunwattana et al.^14^ designed a two-staged CNN-based cell detection and classification algorithm, which has been utilized by various approaches.^15^^,^^16^ These incorporated graph structures to represent cell communities and thereby created phenotypic signatures. By splitting WSIs into smaller patches and mapping each to their most similar phenotypic signature, a multiclass WSI cartography could be created. On colorectal cancer specimens, Sirinukunwattana et al.^15^ scored an accuracy of 97.4% averaged over nine tissue classes and Javed et al.^16^ an $F_{1}$ score of 92% averaged over six classes. These high classification scores, however, were achieved at the expense of high computation times of up to 50 min per WSI for cell detection and classification.^14^

In the field of texture-based segmentation approaches, Signolle et al.^17^ proposed a method that incorporated several binary hidden wavelet-domain Markov tree classifiers whose outputs were combined using majority voting. The authors scored a class-averaged recall of 71.02% on five tissue classes on ovarian carcinoma specimens with an inference time of up to 300 h per WSI. Other texture-based methods grouped pixels into coherent regions, which were classified using texture-based feature representations. On prostate specimens, Gorelick et al.^18^ achieved a class-averaged recall of 83.88% on eight tissue classes with an inference time of 2 min per 300 × 300 pixel sized patch. Apou et al.^19^ segmented breast cancer WSI into six classes and achieved a class-averaged sensitivity of 55.83% and a class-averaged specificity of 91.4%. The authors stated inference times of under 2 h per WSI.

Due to the high variations in hardware resources and annotation quality, it is often difficult to compare image analysis algorithms in terms of classification accuracy and computational costs. Kather et al.^20^ presented a publicly available dataset of histopathological image data and compared the performance of state-of-the-art image analysis algorithms. Using eight classes, the authors scored a maximum accuracy of 87.4%.^20^ Rachapudi and Devi^21^ used this dataset to train a CNN classifier and scored a class-averaged recall of 79.5% and a precision of 80.13%. Both, Kather et al. and Rachapudi and Devi, however, achieved their quantitative results on test images that completely belonged to one class, and the results are therefore difficult to compare with the performance measures obtained on WSIs with multiple tissue classes present.

Using a binary cartography of histopathological images, primary tumor areas can be defined. Tumor here is defined as a combination of viable tumor cells, interconnecting tumor stroma, and desmoplastic stroma as well as comprised necrotic areas and mucus. A robust definition of the tumor area can provide the basis for automatically evaluating pathological criteria such as tumor extend, composition, or grading. Recent publications achieved tumor Dice scores of 69%^22^ and 75.86%^23^ on breast specimens and 78.2%^11^ on colon samples. A good trade-off between refined segmentation results and low computational complexity was achieved by Guo et al.^24^ who scored a tumor intersection over union (IoU) value of 80.69% at an average inference time of 11.5 min per WSI.

### 2.3 Superpixel Classification

Due to their large size, digitized microscopic images can challenge standard machine learning algorithms. Aiming to reduce computational complexity, a clustering into coherent image segments, e.g., superpixels, has proven advantageous. Zhang et al., for instance, used superpixel clustering to compute a probability map for nuclei presegmentation, which was used as auxiliary input to the subsequent tissue classification network.^25^ Nguyen et al. directly segmented breast tissue samples into coherent tissue regions using a graph-based superpixel algorithm.^26^ The authors, however, merely performed a segmentation and did not infer labels for the computed superpixels. Other existing works manually extracted handcrafted superpixel feature vectors which were then classified using machine learning-based classifiers and thereby enabled a binary^22^^,^^27^ or multiclass^12^^,^^18^^,^^19^^,^^28^ semantic segmentation of medical images. On histological image data, this approach has facilitated the binary segmentation of WSIs in 20 to 45 min by Bejnordi et al.^27^ and up to 60 min by Balazsi et al.^22^ with good performance results indicated by Dice scores of 92.43%^27^ and 69%,^22^ respectively. Mehta et al.^12^ segmented breast cancer tissues into eight classes using superpixels and a support vector machine (SVM) for classification. Since this combination was not the focus of their work, but merely served as a baseline for performance comparison of their proposed method, the usage of superpixels has not been evaluated in much detail. Zormpas-Petridis et al.^28^ applied a combination of superpixels and SVM-based classification on the task of segmenting melanoma WSIs. Their evaluation, however, was carried out with a randomly chosen set of superpixels, i.e., the ground truth did not contain the entire annotated tissues as in our work.

Considering the classification of image data, there has been a trend toward the use of deep learning methods, specifically CNNs, in recent years. Bianconi et al.^29^ provided a comprehensive overview from theory-driven (handcrafted) to data-driven (deep-learning) color and texture descriptors. Tamang et al.^30^ summarized various deep learning-based and classical approaches especially for the application of colorectal cancer diagnostics. One significant advantage of deep learning is that it enables a closed-form optimization of classification problems whereas classification based on handcrafted features typically requires the selection of the most characteristic features followed by optimization of the classifier. In addition, CNNs often achieve more accurate classification results than traditional methods, especially when large amounts of labeled data are available for training, which was also shown in a comparison of different approaches for the classification of Malaria pathogens in microscopic image data made by Krappe et al.^31^

Due to their irregular size and shape, however, superpixels can challenge CNN classifiers that require square input images of predefined size. Previous work in the field of histopathology can be categorized into two basic strategies to overcome this issue. The first group of approaches^32^^–^^35^ extracted bounding boxes around superpixels and resized them to a predefined input size. This strategy either requires equally sized superpixels to maintain a similar downscaling factor for all superpixels or loses proportions across the input images. The latter can lead to ignoring the valuable size property of biological structures, e.g., the typically enhanced size of tumor cells, which can be an indicator for neoplastic growth. The second group of approaches^36^^,^^37^ classified a precomputed superpixel by extracting a patch with predefined size around the centroid of the superpixel. These approaches, however, relied on compact and square-like superpixels. Otherwise, the centroid might not lie within the given superpixel and the extracted patch will not be representative of this superpixel. Biological structures, however, are rarely square-shaped and especially at tumor boundaries the interaction of tumor, healthy tissue, and inflammatory or necrotic reactions can lead to very irregularly shaped superpixels. To meet these characteristics of biological tissue and tumor growth, approaches that can be applied to superpixels of varying shapes and sizes are highly desired. Moreover, all of these approaches^32^^–^^37^ relied on a one-to-one relationship between superpixel and the corresponding image patch, which is classified or processed by a CNN. Only Pati et al. subsequently merged neighboring and similar superpixels and averaged their CNN feature vectors to use them in their tissue graph. In our approach, however, the superpixel shape is allowed to deviate greatly from a square shape, and the size of the superpixels is on average 20 times larger than the size of the image patches which are classified by the CNN. This opens up the possibility of classifying multiple image patches within a superpixel and combining patch classification results to a superpixel label through majority voting. Moreover, this one-to-many relationship between superpixel and image patches allows deducing a classification confidence measure from the individual patch classification results.

## 3 Material and Methods

The proposed image analysis pipeline has been trained and evaluated on colon WSIs, provided by the Institute of Pathology of the University Hospital Erlangen (UKER). In the following sections, an overview of the datasets and a detailed description of the applied methods is given.

### 3.1 Datasets

**Fig. 1:**
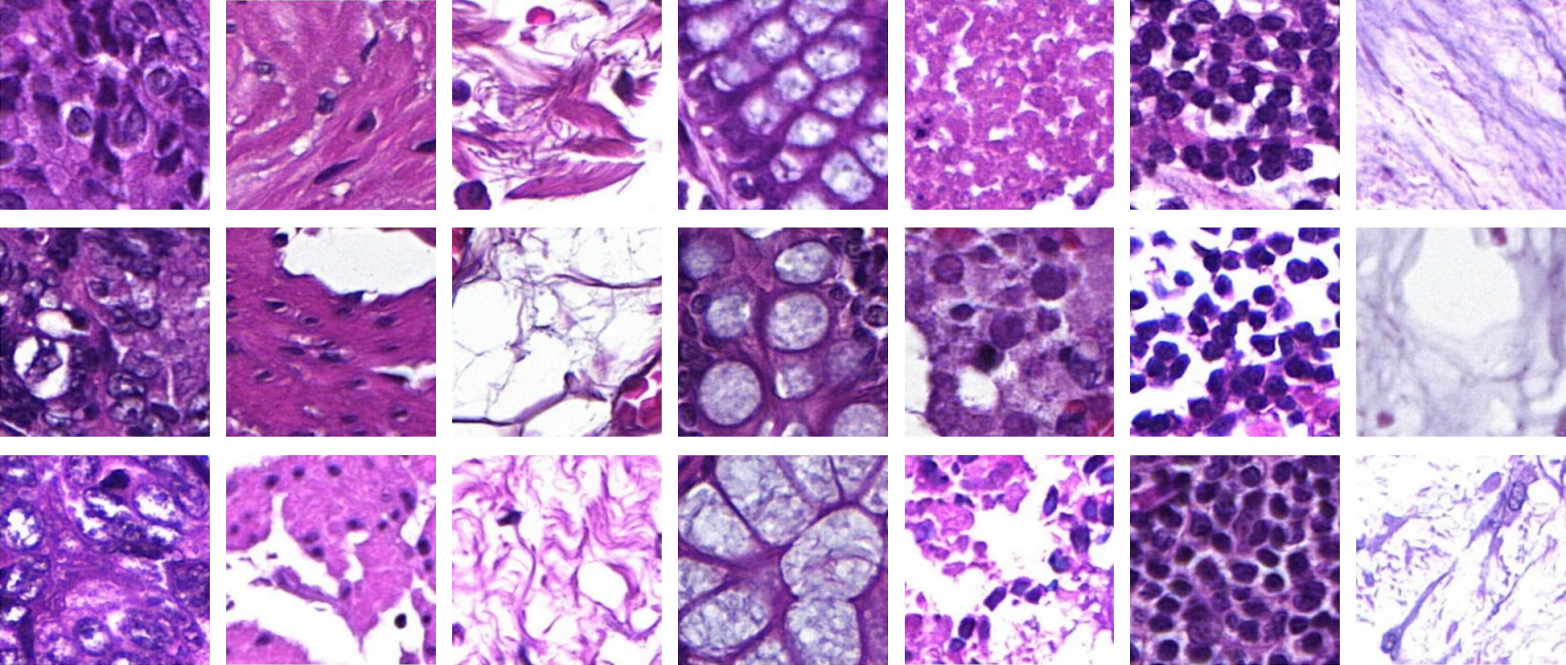
In each column representatives for one tissue class are displayed. From left to right: tumor cells, muscle tissue, connective tissue combined with adipose tissue, mucosa, necrosis, inflammation, and mucus.

For this work, two different datasets have been used. Dataset A comprises 159 annotated H&E-stained WSIs. The microscopic slides were digitized using a 3D HISTECH Pannoramic 250 slide scanner with an objective magnification of 20× and a resolution of 0.22×0.22  μm/pixel. Pathologist-approved manual annotations cover seven tissue classes: tumor cells, muscle tissue, connective tissue combined with adipose tissue, mucosa, necrosis, inflammation, and mucus. Figure 1 visualizes three representatives of each annotated class. Based on these annotations, patches of a size of 224×224  pixels that were covered to at least 85% by one annotation class have been extracted and labeled accordingly. These patches have been used for training and validating a neural network for semantic image segmentation. Table 1 provides an overview of the dataset including the total number of patches and the corresponding area.

**Table 1:** Overview of dataset A. The parameter test set is used for superpixel configurations.

	# Slides	# Patches (224×224)	Area (${\mathrm{mm}}^{2}$)
Training set	92	2,173,515	5278
Validation set	30	719,010	1746
Parameter test set	8	—	612
Test set	29	—	3047
Sum	159	2,892,525	10,683

A second dataset (dataset B) has been used for answering medical research questions, including tumor area estimation and composition. This dataset comprises 18 H&E-stained samples with annotations of the primary tumor area and necrosis, inflammations and mucus within. In addition, for each sample, an AE1/AE3 antibody immune histochemical staining (IHC) as described by Pour Farid et al.^38^ on a consecutive serial section was available.

The retrospective study was approved by the scientific committee (CCC – tissue biobank) of the Comprehensive Cancer Center (CCC Erlangen-EMN; application-No. 100030; date of approval May 9, 2012) of the Friedrich-Alexander University Erlangen-Nuremberg. The study is based on the approval of the Ethics Commission of the University Hospital Erlangen (No. 4607 from January 18, 2012). The study is in accordance with the declaration of Helsinki and ethical guidelines applicable for retrospective studies were respected for all experiments. Tissue histology was reviewed by two pathologists. Pathology reports and medical records of patients who underwent an operation at our hospital were reviewed.

### 3.2 Image Analysis Pipeline

The developed image analysis pipeline is designed as a twofold approach: first, the WSI is segmented into superpixels using the simple linear iterative clustering (SLIC) algorithm.^39^ Then, each superpixel is classified using a CNN-based approach.

#### 3.2.1 Superpixel segmentation

With the goal of reducing the computational complexity of a pixel-based clustering algorithm, the input WSI is analyzed at a coarser resolution level (3.54  μm×3.54  μm/pixel) corresponding to a downscaling factor of 16 in each dimension with respect to the original resolution. Moreover, the WSI is cropped at the tissue’s bounding box. The foreground (tissue) is determined by applying a simple intensity threshold to identify white background pixels (3.54  μm×3.54  μm/pixel resolution). Afterward, the remaining input image is segmented into superpixels. We compared different established superpixel clustering algorithms by Achanta et al.,^39^ Beucher,^40^ Felzenszwalb and Huttenlocher,^41^ and Vedaldi and Soatto.^42^ These experiments demonstrated the superiority of the SLIC algorithm regarding boundary detection of different tissue types and computational efficiency, which is in correspondence with the observations by Achanta et al.^39^ In this work, we employ the SLIC implementation from the Python scikit-image module. To utilize prior knowledge about the histological staining (H&E), a color deconvolution^43^ is performed on the input image and the SLIC algorithm has been modified by replacing the clustering in $[l,a,b,x,y{]}^{T}$-space with a clustering in $[H,E,x,y{]}^{T}$-space. To avoid overly jagged contours, the image is smoothed prior to segmentation using a Gaussian filter (σ=5). The SLIC’s number of k-means iterations is limited to 10. The average superpixel size is set to 3600 pixels at the downscaled resolution level (i.e., a square superpixel would cover $0.2\times 0.2\;{\mathrm{mm}}^{2}$). This average superpixel size was determined on a subset of dataset A, which was solely used for parameter configuration (see Table 1, Sec. 4.1). Accordingly, the input parameter for the number of superpixels to be generated by the SLIC algorithm is set to

$$k=\frac{\mathrm{pixelCount}(\mathrm{boundingBox}(\mathrm{foreground}(\mathrm{WSI})))}{3600}.$$ (1)

After segmentation, all superpixels that contain at least 50% white pixels are labeled as background. These superpixels are excluded from any subsequent classification. The threshold of 50% has been set as a compromise to achieve an accurate tissue-background separation while not disregarding superpixels that cover adipose tissue, which oftentimes also contains large white areas.

#### 3.2.2 Superpixel classification

**Fig. 2:**
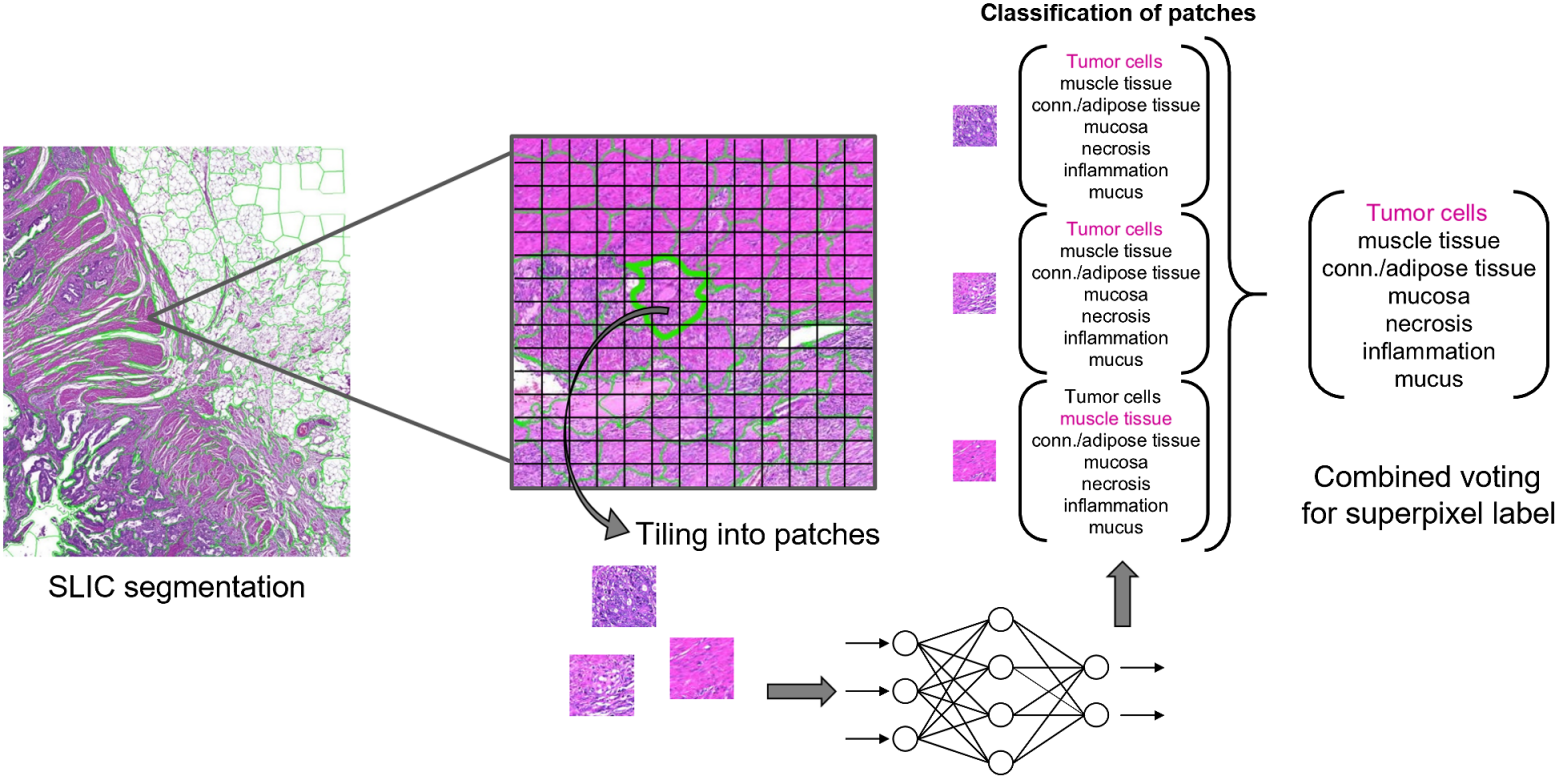
Superpixel classification workflow.

Figure 2 visualizes the algorithm for inferring the superpixel class-labels. Initially, the input image is divided into equally sized, nonoverlapping patches of 224 × 224 pixels at the original image resolution of 0.22×0.22  μm/pixel (20×). Afterward, all patches covered by at least 50% of one superpixel are classified using a CNN. By lowering this threshold, the absolute number of patches that account to a superpixel’s classification result increases, but so does the relative number of in-distinctive border patches. The threshold of 50% was found to be a good trade-off during preliminary experiments on the parameter optimization subset of dataset A. After patch classification, all patch labels are combined to infer a superpixel classification. Various standard CNN architectures utilize a softmax layer to output a probability distribution over all classes. We propose to compute the combined probability distribution by summing up over all patch softmax output vectors and normalizing by N, the number of patches that contribute to the classification result. The superpixel label $L_{\mathrm{SP}}$ is then defined by the class corresponding to the maximum entry in the superpixel’s probability distribution:

$$L_{\mathrm{SP}}=\underset{c_{i}}{\arg\max}(\frac{\overset{N}{\underset{n=1}{\sum }}[p_{n,c_{1}},p_{n,c_{2}},\ldots ,p_{n,c_{k}}{]}^{T}}{N})\;=\underset{c_{i}}{\arg\max}(\frac{\overset{N}{\underset{n=1}{\sum }}{\overset{\to }{p}}_{n}}{N}),$$ (2)

Here, $c_{i}\in C$ is the set of available class-labels.

Preliminary experiments have shown that due to a high variance in shape and size of the superpixels sometimes up to 100 individual patches account to a superpixel label. Since the vast majority of patches within a superpixel will contain the same tissue type, we hypothesize that valuable computation time can be saved by analyzing only a random subset of patches without significantly impacting the overall accuracy. We propose to analyze at most 10 patches. The influence of this restriction is investigated in Sec. 4.1

Moreover, we propose a confidence measure ($C_{\mathrm{diff}}^{\mathrm{votes}}$) of a superpixel classification derived from the classification results of the patches within this superpixel. For this, we divide the difference of patch votes for the most represented and the second most represented class by the number of all patches.

#### 3.2.3 CNN model

The developed preprocessing steps (color deconvolution, foreground detection, and superpixel segmentation) are independent of the subsequent CNN structure, which is therefore interchangeable. However, the average superpixel size has to be adapted to the CNN input patch size to maintain a reasonable ratio of both measures. For the experiments elaborated hereafter, a ResNet50 architecture with 224×224  pixel input size has been chosen and trained using training and validation set of dataset A (see Table 1). The network has been implemented using TensorFlow 2.2. We employ the color augmentation method described by Tellez et al.,^44^ where the RGB image is converted to the H&E color space using a deconvolution. Then, the H&E components are individually modified, simulating different staining intensities. Moreover, zero-centering is applied as a preprocessing step. Training is performed using cross entropy loss and Adam optimizer with a learning rate of 0.001. A batch-size of 105 was chosen and in each batch the different classes are represented equally. Class imbalances are hereby compensated by oversampling of underrepresented classes as for example necrosis and mucus.

### 3.3 Evaluation Method for Cartography Results

For a visual validation of the annotation ground truths and classification outputs, the Open Source software tool SlideRunner^45^ has been used. The quantitative analysis is performed with an image resolution of 3.54  μm×3.54  μm/pixel. We assign a class-label to each foreground pixel according to the manual ground truth annotation. The prediction map of the image is generated with the same resolution. Only pixels having both a ground truth and a prediction label are evaluated. Based on the confusion matrix, different classification measures such as, e.g., classwise recall are calculated. For all classwise measures, the corresponding two-class problem is regarded whereby all negative classes are combined to one class.

### 3.4 Tumor Area Computation and Invasive Margin

The primary tumor area is determined based on the cartography results. First, binary maps for the classes “tumor cells,” “necrosis,” and “mucus” at the same resolution level used for superpixel segmentation (3.54  μm×3.54  μm/pixel) are created. A morphological closing operation is applied to the “tumor cells” map and each connected component of the necrosis and mucus-map is checked for whether it is located adjacent to a tumor cell component. All adjacent necrosis and mucus components and all tumor cells components are added to a tumor map. Afterward, morphological closing followed by opening is applied. Finally, the tumor area is given by summing up areas enclosed by the outer contour of each tumor component. Besides the direct comparison of the calculated area (E) and the annotated ground truth area (GT), the IoU and Dice coefficient metrics are computed:

$$\mathrm{IoU}=\frac{\left|E\cap \mathrm{GT}\right|}{\left|E\cup \mathrm{GT}\right|}=\frac{\mathrm{TP}}{\mathrm{TP}+\mathrm{FP}+\mathrm{FN}},$$ (3)

$$\mathrm{Dice}=\frac{2|E\cap \mathrm{GT}|}{\left|E|+|\mathrm{GT}\right|}=\frac{2\mathrm{TP}}{2\mathrm{TP}+\mathrm{FP}+\mathrm{FN}},$$ (4)

$$\mathrm{TP}:\mathrm{true}\;\mathrm{positives}\text{-}\mathrm{pixel}\;\mathrm{is}\;\mathrm{contained}\;\mathrm{in}\;\mathrm{both}\;E\;\mathrm{and}\;\mathrm{GT};\;\mathrm{FP}:\mathrm{false}\;\mathrm{positives}\text{-}\mathrm{pixel}\;\mathrm{is}\;\mathrm{contained}\;\mathrm{in}\;E\;\mathrm{but}\;\mathrm{not}\;\mathrm{in}\;\mathrm{GT};\;\begin{array}{c} \mathrm{FN} \end{array}:\mathrm{false}\;\mathrm{negatives}\text{-}\mathrm{pixel}\;\mathrm{is}\;\mathrm{contained}\;\mathrm{in}\;\mathrm{GT}\;\mathrm{but}\;\mathrm{not}\;\mathrm{in}\;E.$$

The tumor invasive margin is derived from the autodetected tumor area by extending the region in relation to the desired margin width. The intersection between this extended region and nontumor tissue defines the basis of the invasive margin. Finally, the intersection area is again extended as the invasive margin is situated at the border between tumor and surrounding tissue and stretching out into both.

### 3.5 Tumor Composition

Quantitative analysis of the tumor microenvironment supports studies and diagnostics of tumor-infiltrating lymphocytes in colon, as well as bladder and breast cancer.^46^^,^^47^ The analysis in a region of interest such as the invasive margin plays an important role for evaluating the immune response against tumor cells. In previous studies, a high correlation between CD3- and CD8-positive cell counts and patient outcome has been shown.^48^ In the case of colon cancer, the immune response can be quantified using the immunoscore.

**Fig. 3:**
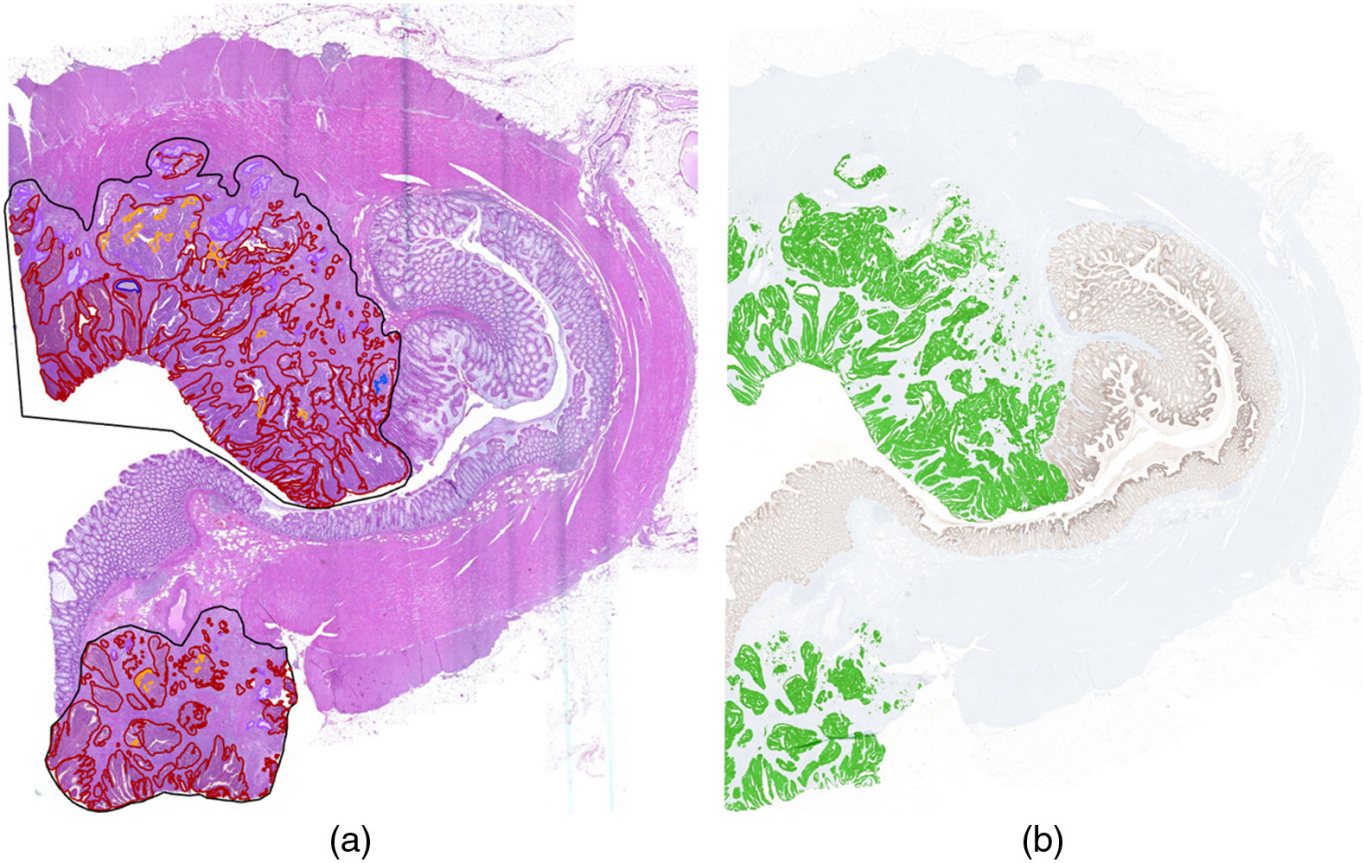
(a) Manual annotation of tumor (black), necrosis (orange), and mucus (purple) in H&E-stained colon section. (b) Active tumor area (green) in corresponding IHC-stained colon section.

Therefore, we use dataset B to compare the estimation of tumor component areas (active tumor, necrosis, mucus) from cartography results with the manual annotations. Graham et al.^49^ used a rotation equivariant network for the task of gland segmentation. We use this approach to separate the active tumor area from interconnecting tumor stroma using the segmented glands’ area as approximation for the active tumor area. The ground truth area for necrosis and mucus is directly derived from the manual annotations. The ground truth for the active tumor area is obtained on serial sections stained with immunohistochemical markers (pan-cytokeratin, epithelial AE1/AE3) by applying a simple thresholding approach within the manually annotated tumor area. Again, color deconvolution was performed and solely the DAB channel was chosen for segmentation. Figure 3 shows a comparison of the manual annotations on the H&E-stained WSI and the segmentation result on the IHC-stained consecutive WSI. On the one hand, this approach is beneficial as it does not suffer from the human annotator’s subjectivity. On the other hand, one has to keep in mind there is a small spatial distance between the two consecutive sections that is large enough that a cell visible in one slide might not be visible in the other slide.

## 4 Results and Discussion

Several experiments were performed using dataset A to investigate the performance of the superpixel-based WSI cartography. Starting from parameter configuration of the SLIC algorithm, followed by a comparison between a classical patch-based approach with our newly introduced superpixel approach up to an investigation of uncertainty of the superpixel classification results. Though not the focus of this work, we carried out preliminary experiments on dataset B for two possible medical endpoints (tumor area and tumor composition) that will likely benefit from having a detailed tissue map available as it is generated by our proposed method.

### 4.1 Configuration of Superpixel Approach

To define an optimal average superpixel size as well as a threshold for the number of classified patches per superpixel, experiments were performed on the parameter test set of dataset A containing eight WSIs (see Table 1).

**Fig. 4:**
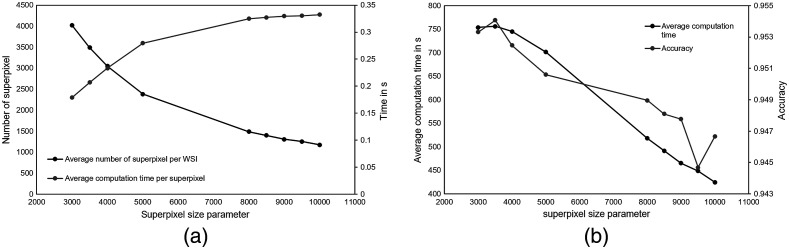
(a) Influence of average superpixel size on the average number of superpixels per WSI and the average computation time per superpixel classification and (b) the average computation time and accuracy for inference on the overall slide. Evaluations were performed on the parameter test set of dataset A and the maximum number of classified patches per superpixel was limited to 30.

Figure 4 visualizes the influence of increasing the average superpixel size (as start parameter for the SLIC algorithm) on the total number of superpixels per WSI, the classification accuracy, and the average computation times for superpixel classification and WSI inference. For these experiments, the maximum number of classified patches per superpixel was limited to 30. Inference times have been measured using an NVIDIA GeForce GTX 1060 GPU with 6 GB RAM. As expected, a larger average size per superpixel results in fewer superpixels per WSI. However, larger superpixels cover a larger number of patches, which are classified and then combined to infer a superpixel class-label. Therefore, larger superpixels entail higher computational costs [Fig. 4(a)]. Nevertheless, the overall computation time for slide inference decreases due to the decreased number of superpixels on the WSI. The classification accuracy, however, also decreases [Fig. 4(b)].

**Fig. 5:**
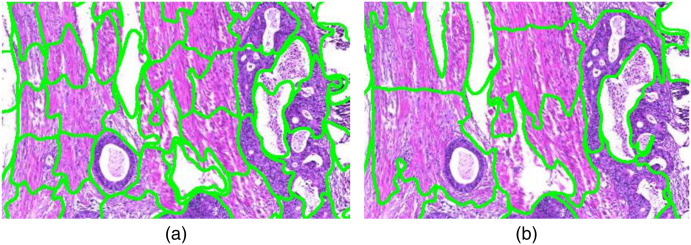
(a) Superpixel segmentation result with size of 3.600 pixels compared to (b) the segmentation result with a size of 10.000 pixels. The segmentation results on the left side fits better to the tumor cell boundaries but in both images necrotic areas within the tumor are not always detected as separate regions.

Figure 5 visualizes the effect of smaller superpixel sizes [Fig. 5(a)] and larger superpixel sizes [Fig. 5(b)] on the segmentation result. As compromise between low computational complexity for larger superpixel sizes and high accuracy for smaller superpixel sizes, an average superpixel size of 3600 pixels, i.e., a square superpixel would cover $0.2\times 0.2\;{\mathrm{mm}}^{2}$, was chosen for further experiments. However, the results of these experiments depend on various parameters (such as the threshold for the number of classified patches per superpixel) as well as the chosen CNN architecture, and there is still room for further optimization. The biggest disadvantage of a greater superpixel size is that small details are neglected, resulting in inaccurate segmentation results especially for classes such as necrosis or tumor cells.

**Fig. 6:**
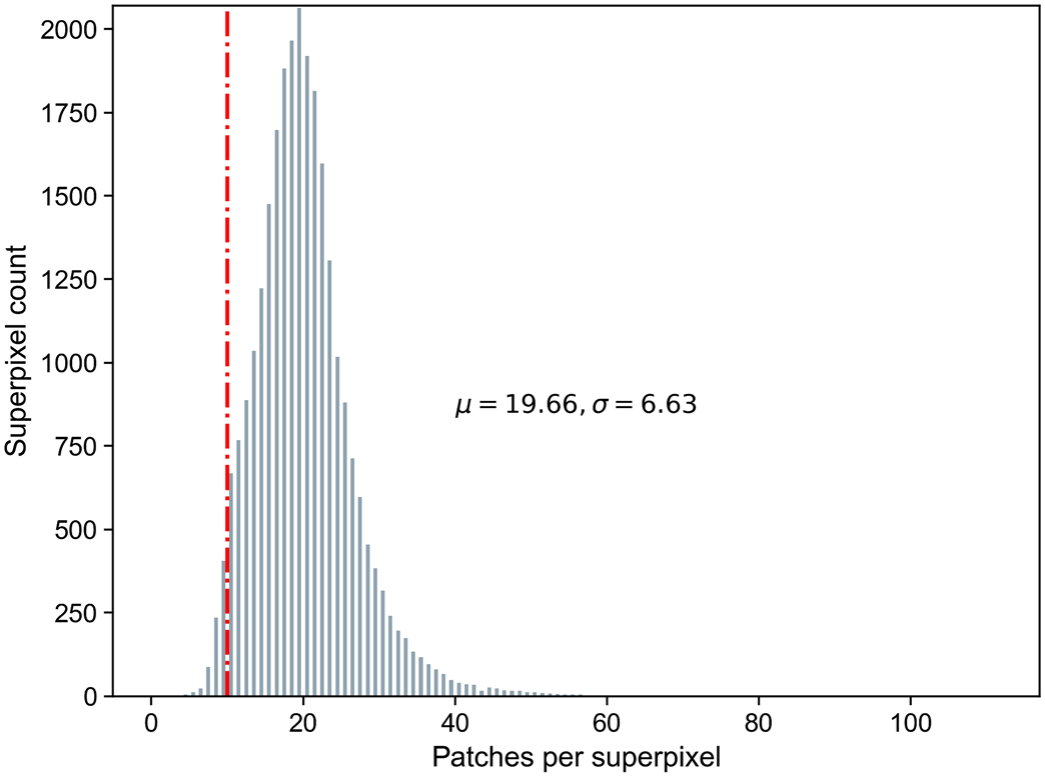
Superpixel count over number of patches per superpixel for an average superpixel size of 3,600 pixels. The histogram sums up all WSIs in the parameter test set of dataset A. On average, a superpixel contains 19.66 patches with a standard deviation of 6.63.

The histogram in Fig. 6 shows that with an average superpixel size of 3600 pixels, some larger superpixels cover more than 30 individual patches. We hypothesized that it is sufficient to only classify a random subset of the patches within a superpixel. Table 2 summarizes the influence of various maximum patch limits on the computation time of slide inference and overall accuracy. While a smaller patch limit results in significantly lower computational costs, the slide accuracy only shows a marginal decrease. Therefore, we further reduced the limit from 30 to 10 patches for subsequent experiments.

**Table 2:** Average computation time and overall accuracy on parameter test set for different limits of number of classified patches per superpixel.

Patch restriction per superpixel	Average computation time per WSI (s)	Overall accuracy (%)
10	408	95.1
18	643	95.3
20	686	95.3

### 4.2 Classification Performance and Run-Time

To evaluate segmentation performance and computational complexity, the proposed algorithm is compared to a traditional classification-based approach with nonoverlapping image patches. To isolate the effects produced by the proposed technique of introducing a superpixel clustering and inferring superpixel classification labels, the same CNN is used as part of both approaches. Results are collected on the remaining 29 slides of dataset A (test set), which have not been used for training, validation, or adaptation of parameters. The classification performance is assessed pixelwise on a lower image resolution of 3.54  μm×3.54  μm/pixel as described in Sec. 3.3. Table 3 summarizes the total number of evaluated pixels on this resolution. Minor deviations of the overall sum of evaluated image pixels exist due to the irregular shape of the superpixels compared to the patchwise approach.

**Table 3:** Number of evaluated pixels (resolution 3.54  μm×3.54  μm/pixel) for the patch-based and superpixel approach. Differences are caused by the background detection and the irregular size of superpixels.

	# Pixels (patch-based)	# Pixels (superpixel)
Tumor cells	61,575,137	61,526,076
Inflammation	2,157,405	2,157,411
Conn./adipose tissue	75,677,369	76,463,057
Muscle tissue	60,759,062	60,652,301
Mucosa	38,708,588	38,654,828
Mucus	620,245	620,823
Necrosis	5,883,073	5,874,393
Sum	245,380,879	245,948,889

On the 29 test slides of dataset A, the tissue bounding box contains on average 10.7 billion pixels on the native resolution ($≙520\;{\mathrm{mm}}^{2}$). Within these, the SLIC algorithm produces 4060 ± 1717 (μ±σ) superpixels with an average size of 1,016,289 pixels ($≙0.05\;{\mathrm{mm}}^{2}$). The average number of patches per superpixel without introducing a maximum cut-off is 19.58 ± 6.39. A restriction of the maximum number of patches to be classified to only 10 patches per superpixel affects 94.8% of all superpixels and decreases the average number of classified patches per superpixel to 9.95 ± 0.36.

**Fig. 7:**
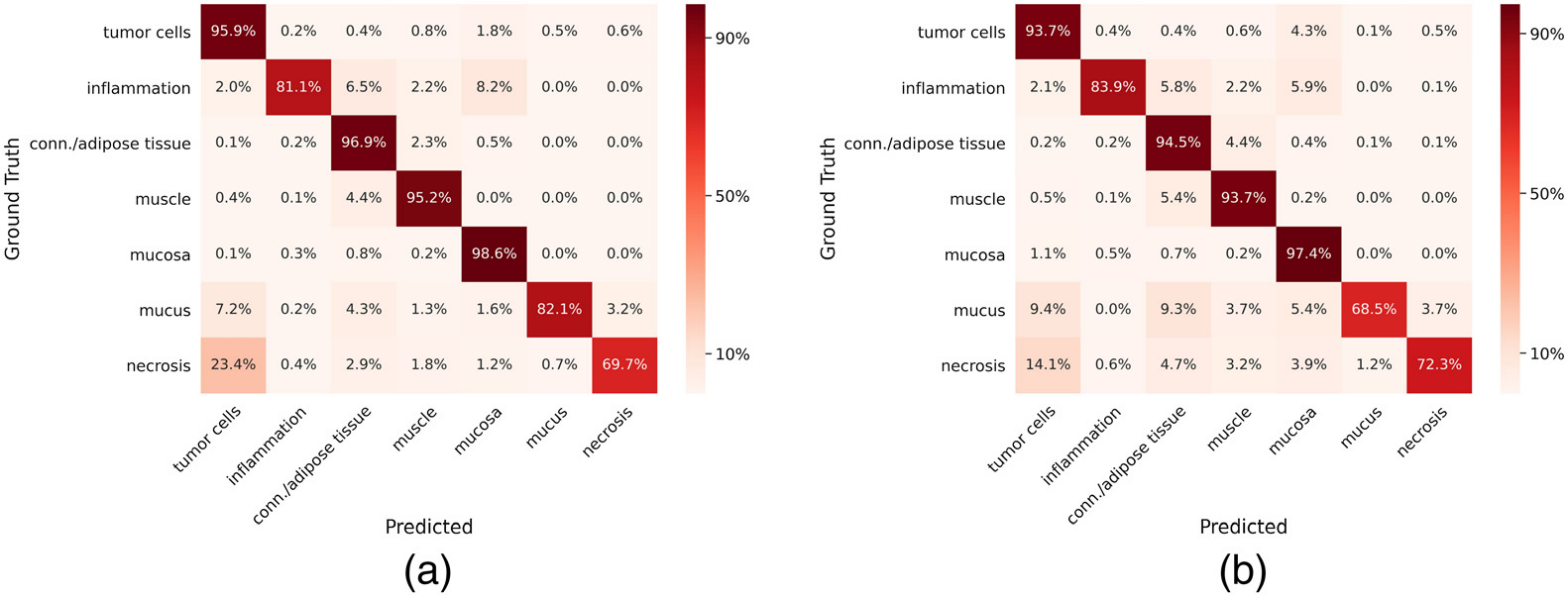
Comparison of confusion matrices of (a) superpixel-based approach and (b) patch-based approach. The rows represent the ground truth class-labels and the columns represent the predictions. Due to high imbalances in the number of pixel per class, a relative representation of the confusion matrix was chosen.

When evaluating a multiclass semantic segmentation task, it is informative to look at which classes are frequently mistaken for one another. Figure 7 shows the relative confusion matrices for both approaches. They show similar behavior regarding the typical confusions of classes: e.g., necrosis is misclassified as tumor or inflammation as mucosa.

**Fig. 8:**
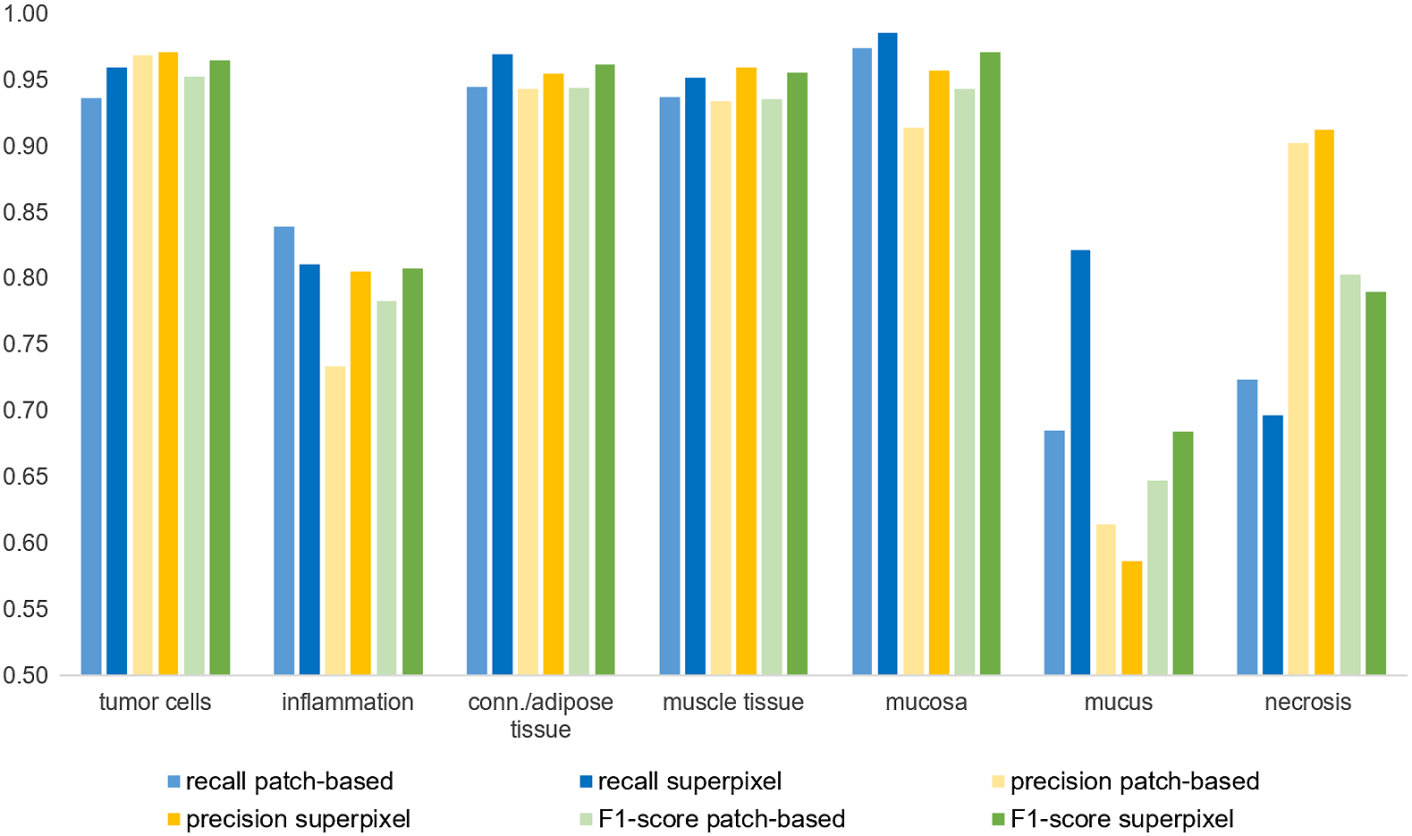
Comparison between patch-based and superpixel-based approach by classwise recall, precision, and $F_{1}$ score.

From the confusion matrices class-based recall and precision values are calculated, which are displayed in Fig. 8. The superpixel-based approach yields an overall accuracy of 95.7% compared to 93.8% obtained with the patch-based approach. The improvement in accuracy has been tested for statistical significance using the two-matched-samples t-test based on the 29 slidewise classification accuracies and has been verified on a confidence interval of 99%. Due to differences in the background detection which is performed per superpixel and respectively per patch, the sum of classified pixels slightly differs between the two approaches. Figure 8 shows an improvement of the classification measures with the superpixel approach compared to the patch-based approach. The average improvement in recall is 0.022, 0.019 for precision, and 0.018 for the $F_{1}$ score. While this improvement can be observed for all classes with larger annotation areas, the performance sometimes decreased for inflamed, necrotic, and mucous areas. One possible reason for this might be that these classes constituted very fine annotations. The chosen superpixel size sometimes creates clusters too coarse to accurately represent these minute structures.

**Fig. 9:**
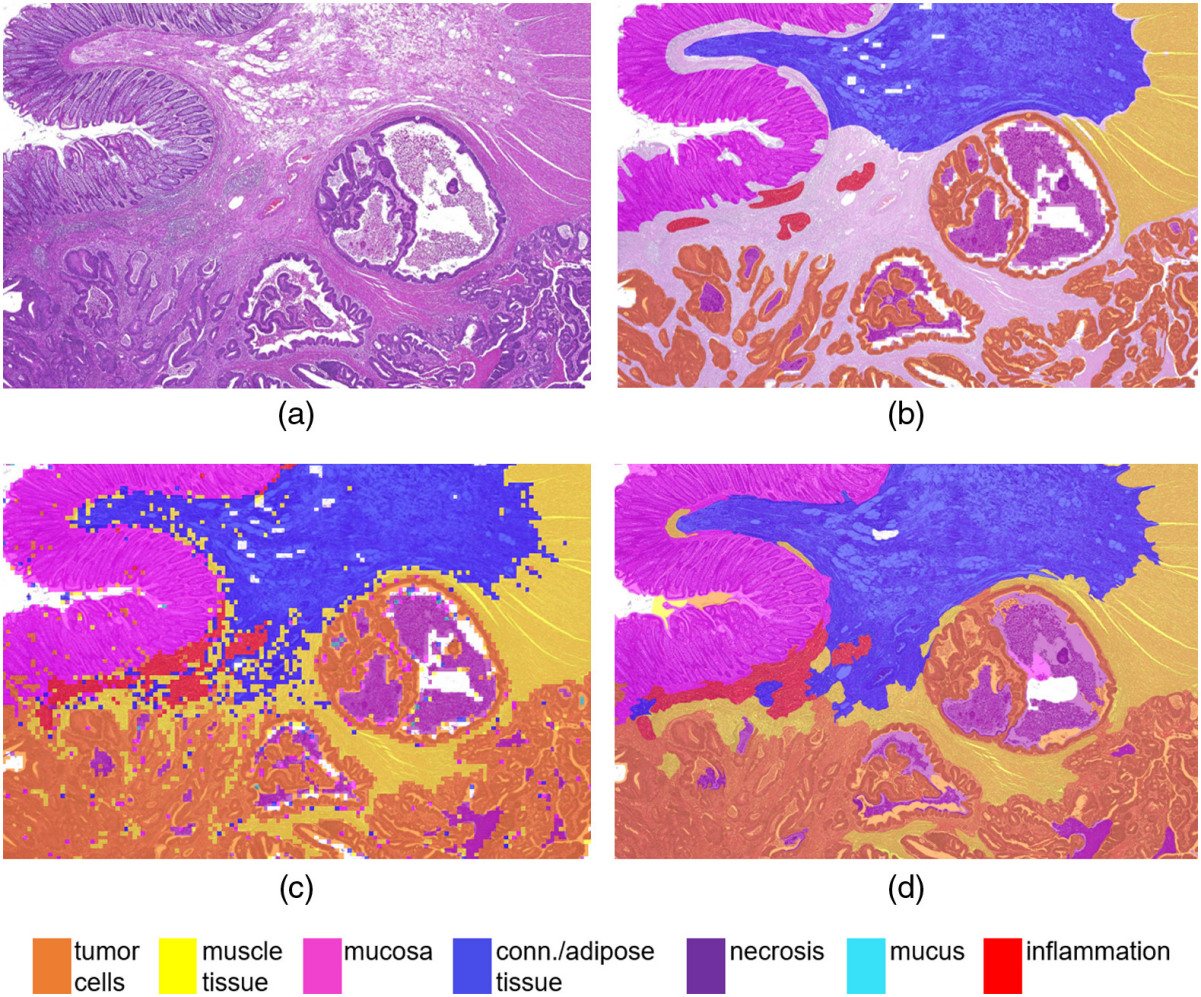
Cartography results: (a) original section, (b) ground truth hand-annotation, (c) patch-based output, and (d) superpixel-based output.

Figure 9 visualizes the cartography outputs of the compared approaches. Overall, the nonoverlapping patch-based image analysis yields checkered classification outputs with many interruptions of connected components due to individual misclassifications. A prior segmentation into superpixels, on the other hand, yields smoother results which follow biological structures. It can be seen that the larger tissue classes are detected accurately and also smaller structures, e.g., inflammations and necrotic areas, are classified correctly in most of the cases. However, this example also highlights limitations of the algorithm, where structures become too small to be accurately represented by the superpixels, e.g., small necrotic areas of comedo necrosis, which is in correspondence with the decrease in recall for necrosis compared with the patch-based approach. This drawback could be countered by choosing a smaller average superpixel size, albeit, only at the cost of higher computation times. The relatively large superpixel size also causes tumor cell classifications to be rather generous and incorporate surrounding tumor stroma. If a precise tumor/stroma separation is intended, the superpixel-based classification approach could be followed by a separate cell-detection-algorithm or simply a second refinement run of the superpixel segmentation and classification restricted to only the tumor areas.

Using an NVIDIA GeForce GTX 1060 GPU and TensorFlow 2.2, the standard classification-based segmentation approach with nonoverlapping patches resulted in computation times of 12.8±5.3  min per WSI. The superpixel-based segmentation pipeline achieved classification times of 6.7±2.8  min with an additional  47±18  s for the SLIC clustering resulting in an overall run-time of 7.5±3.0  min per WSI. Thereby, an average acceleration of 41% could be achieved by the proposed image analysis approach. This acceleration is mainly the result of restricting the number of classified patches per superpixel. Without restriction, the classification time increases to 13.4±5.5  min and the overall run-time including SLIC clustering to 14.2±5.7  min. This is slower than the patch-based approach but yields the highest overall accuracy with 96.0% which is an improvement of 0.3% points compared to the the superpixel cartography with restriction of the classified number of patches per superpixel.

When comparing computation times, it has to be considered that the patch-based approach was performed in the fastest possible way using nonoverlapping patches. Standard patch-based approaches, however, use overlapping image patches and interpolate classification results. When choosing an overlap of half the patch dimension, the number of overall classifications already increases from n×n to (2n−1)×(2n−1). Even when using fast scanning architectures for avoiding redundant computations in overlapping image regions, the overall computational costs are assumed to further increase when using overlaps. This underlines the benefit of the proposed clustering prior to classification even further.

### 4.3 Introduction of Rejection Class Based on Classification Confidence

Aiming to minimize the effect of misclassifications on the final cartography output, we attempt to detect superpixels with uncertain classification results. This way, a rejection label can be assigned to these superpixels. Our hypothesis is that the remaining classification results are more reliable and therefore yield a higher overall accuracy as well as average classwise precision, recall, and $F_{1}$ score. This is done at the expense that unclassified areas are introduced which are not included in the calculation of classification quality measures. Superpixels with a confidence lower than a defined threshold are assigned to the rejection class and hence all pixels (resolution: 3.54  μm×3.54  μm/pixel) inside them as well. All pixels of the remaining superpixels are evaluated as before (see Sec. 3.3). As a consequence of the rejection of unsure pixels, the number of classified pixels and therefore the number of correct and false predicted pixels decreases.

We compared the confusion matrix and classification metrics without and with rejection of uncertain superpixels. As rejection threshold we have chosen 0.1, which means that all superpixels with a $C_{\mathrm{diff}}^{\mathrm{votes}}$ smaller than 0.1 are assigned to the rejection class. In total, 1.3% of the pixels were rejected. The number of total true predictions decreases by 0.8% compared with the classification without rejection while the number of false predictions decreases by 11.8%. Overall, 1.9 million pixels that were correctly classified are discarded due to a low confidence value and 1.3 million pixels that were incorrectly classified. The overall accuracy increases to 96.1% compared with 95.7% without rejection of superpixels. Likewise, there is an improvement for all classes in precision (average 0.009), recall (average 0.007), and $F_{1}$ score (average 0.009). The highest impact is obtained for classes that are usually distributed over the whole tissue sample and cover very small sections such as necrosis, inflammation, and mucus. These results support our hypothesis that the remaining classification results are more reliable at the expense of introducing areas without classification. Therefore, it depends on the application which aspect is prioritized.

**Fig. 10:**
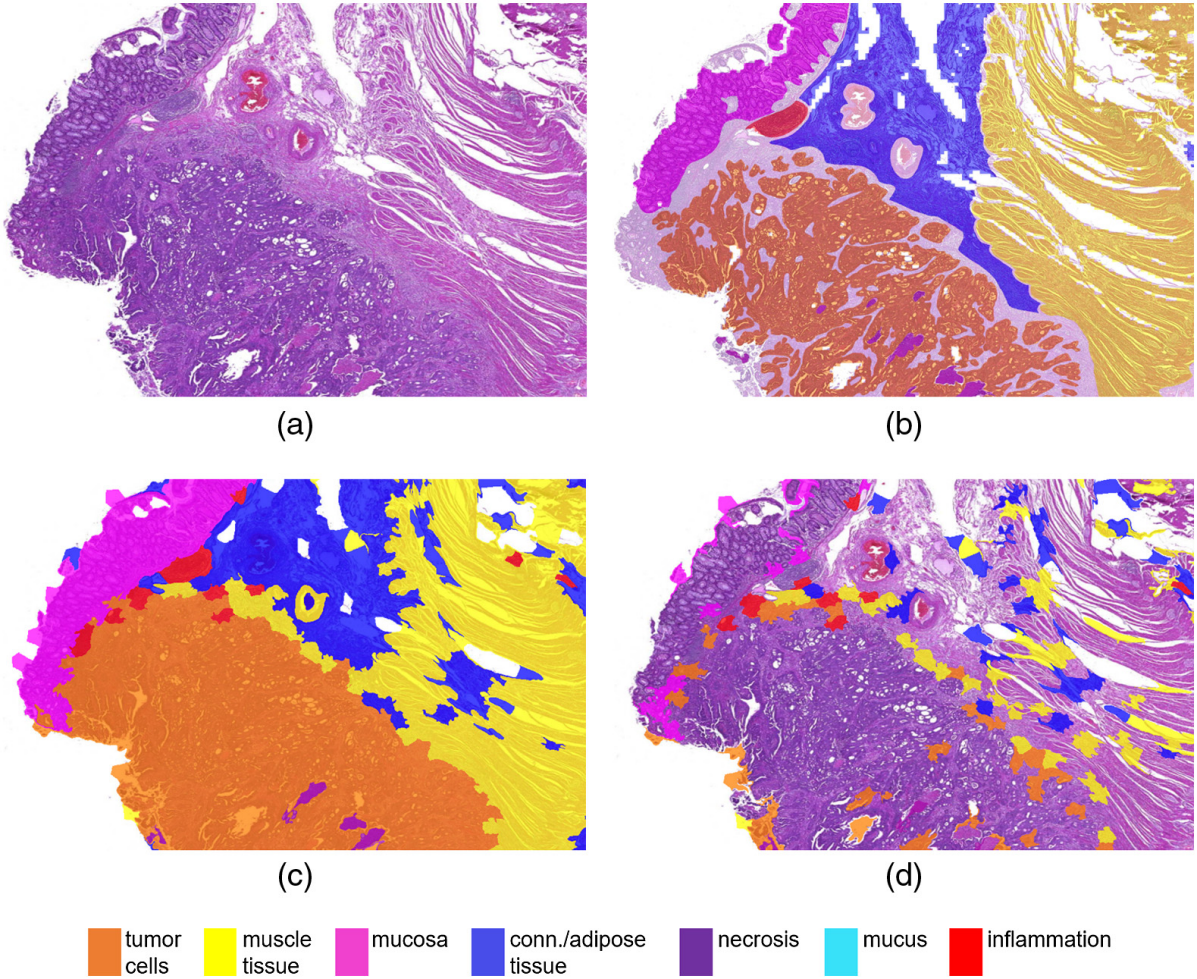
Example for uncertain superpixels based on $C_{\mathrm{diff}}^{\mathrm{votes}}$ with a threshold of 0.45. (a) Original section, (b) ground truth hand-annotation, (c) cartography results, and (d) only superpixels with uncertain classification results are marked. Especially superpixels containing a high amount of background pixels or in the transition of two tissue types tend to show uncertain classifications.

Besides the quantitative evaluation, the question arises about which areas in a WSI tend to achieve uncertain classifications. We only touch upon this question with one qualitative example: In Fig. 10(d), superpixels with uncertain classification results (based on $C_{\mathrm{diff}}^{\mathrm{votes}}$ with a threshold of 0.45) are highlighted. This example reveals two typical constellations that lead to an uncertain classification. Superpixels containing a high amount of background pixels, e.g., located at or nearby fissures or at the rim of the tissue section, tend to be misclassified. The same applies to superpixels in the transition of two tissue types, e.g., located near the invasive margin or slightly inflamed tissue. Moreover, ground truth annotations are only provided for regions that can be assigned clearly to one tissue type except for the tumor cell class. Here, it was not feasible to annotate each small necrotic area which is shown in Fig. 10(b).

### 4.4 Tumor Area

**Fig. 11:**
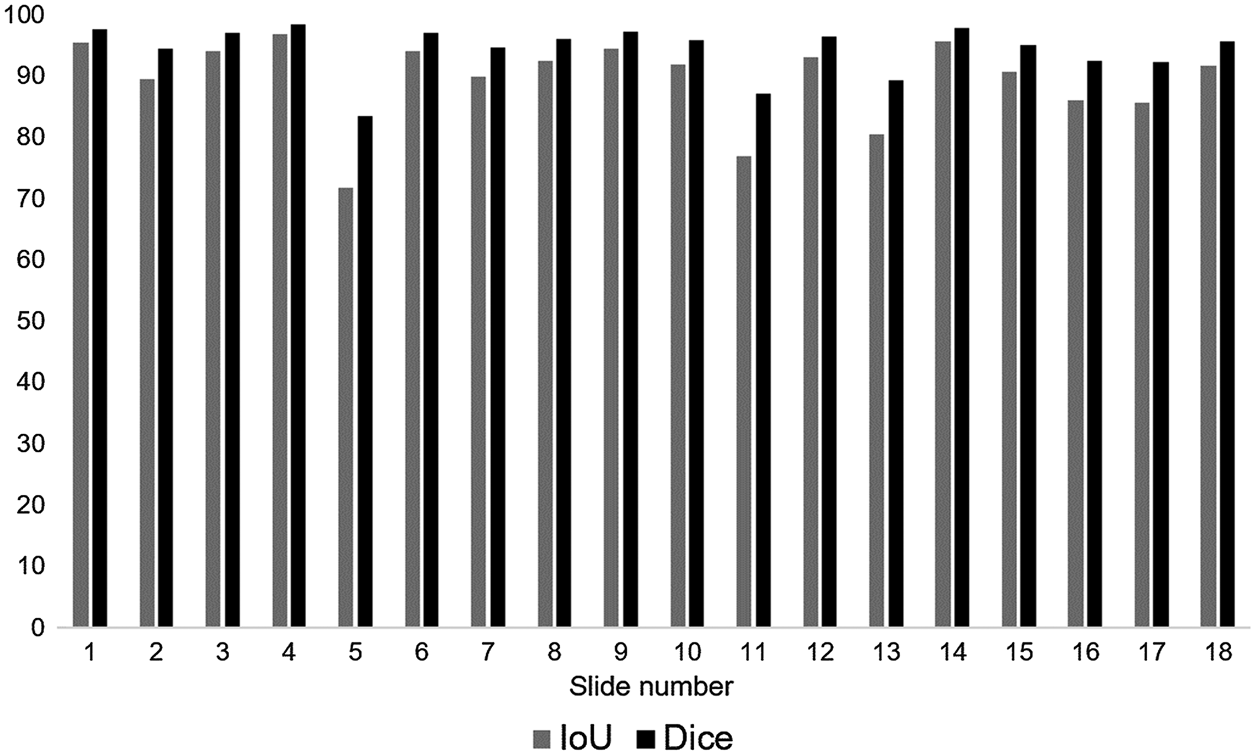
IoU and Dice measure of estimated and annotated tumor area for all 18 slides of dataset B.

Dataset B was used to evaluate the computation of the tumor area. On average, the estimated and the annotated tumor area differ by 6% with a mean IoU of 89.4% and a mean Dice coefficient of 94.3% (per slide results in Fig. 11).

**Fig. 12:**
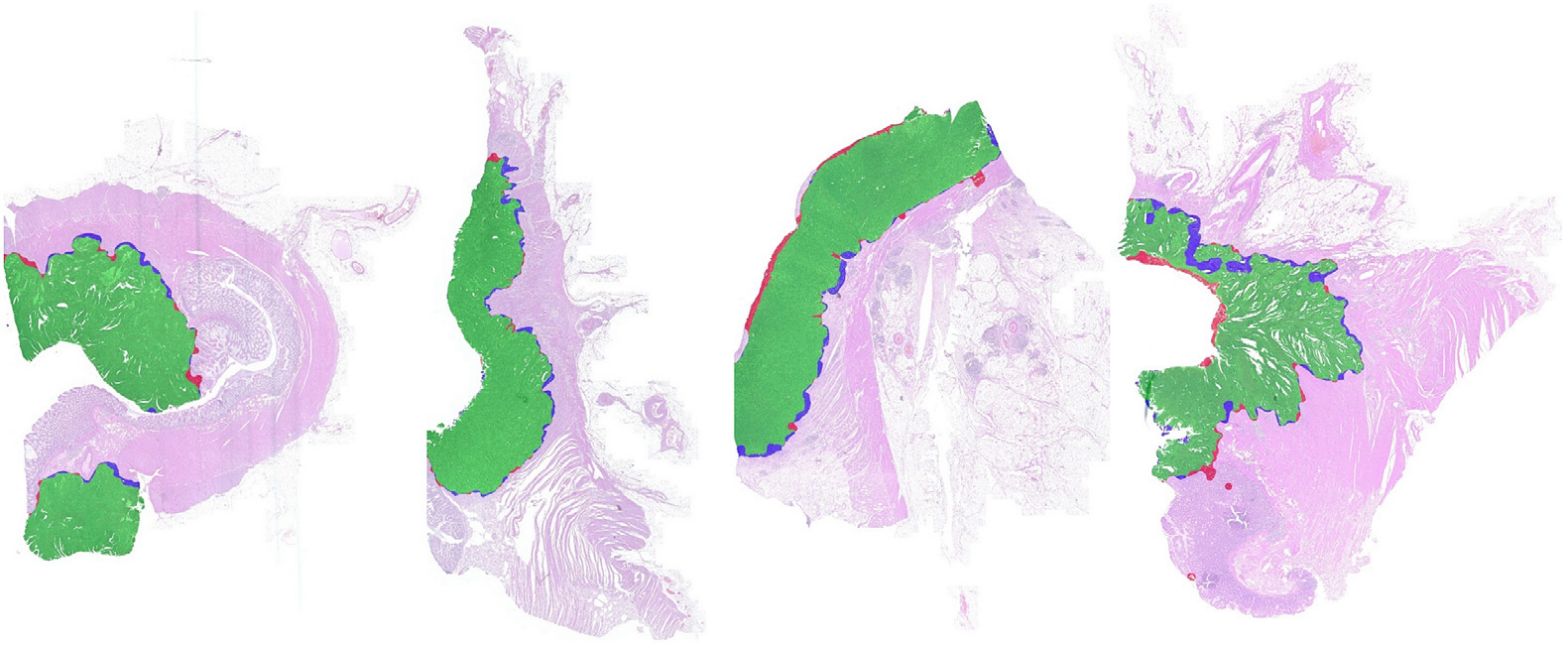
Comparison of tumor area [from left to right: slide numbers 1, 3, 8, 16 (see Fig. 11)]. Green: areas correctly identified as tumor (TPs); red: areas mistaken as tumor (FPs); blue: tumor tissue missed by the classifier (FNs). Misclassifications are largely located at the tumor boundary.

Figure 12 depicts examples of evaluation results, where green overlays resemble tumor areas that have been found correctly (TPs), red marks areas that were mistaken as tumor (FPs), and blue indicates tumor annotations not detected by the algorithm (FNs). It can be seen that most misclassifications are located at tumor boundaries. Especially, necrotic areas adjacent to the lumen were included in the tumor area for our approach but have been excluded by the pathologist. On the contrary, at the invasive margin our approach misses some tumor areas.

**Fig. 13:**
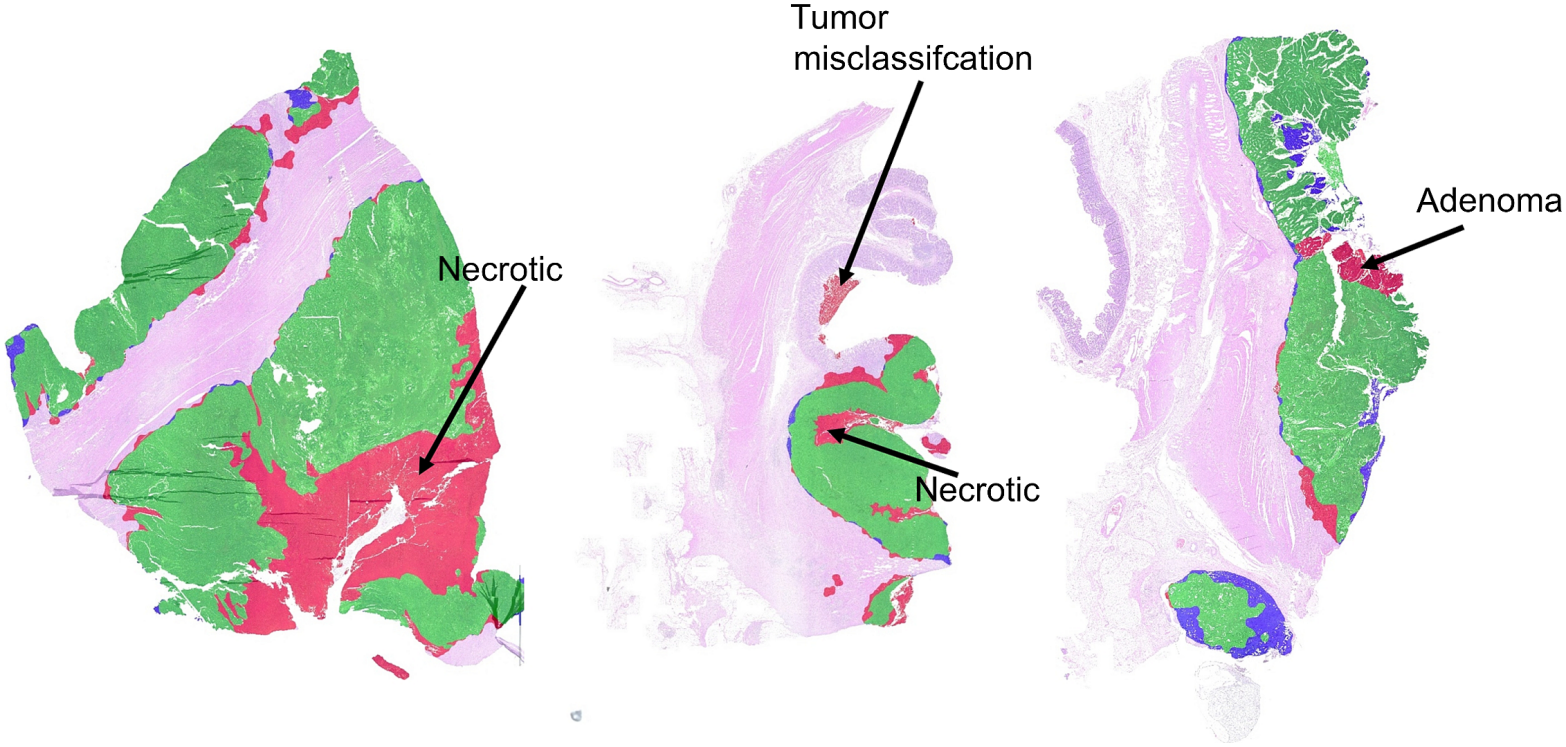
Comparison of estimated and annotated tumor area showing the examples with the highest deviations [from left to right: slide numbers 5, 11, 13 (see Fig. 11)]. Green: areas correctly identified as tumor (TPs); red: areas mistaken as tumor (FPs); blue: tumor tissue missed by the classifier (FNs).

**Fig. 14:**
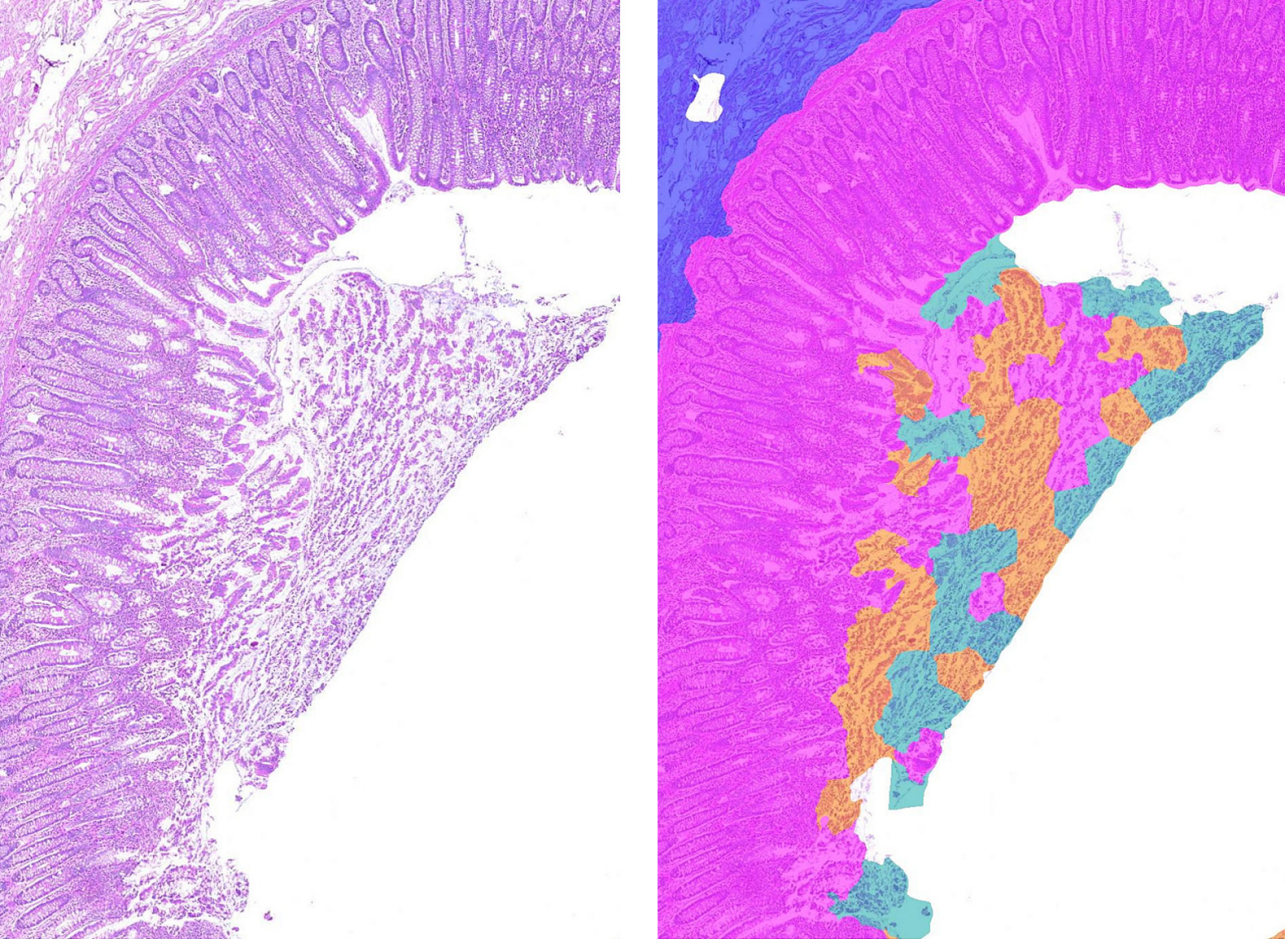
The mixture of debris and destroyed mucosa tissue is classified as tumor (orange) and mucus (turquoise) and leads to a deviation in tumor area in slide number 11.

Looking at the slide results in detail, however, a few WSIs contain larger misclassified regions. Three examples are visualized in Fig. 13. One main source for deviations are again necrotic areas. In our approach, all adjacent necrotic areas are incorporated into the tumor area. This technically defined rule cannot perfectly represent the pathologist’s annotation (ground truth) in individual cases, as it cannot sufficiently reflect the biological and complex morphological nature of the tumor. Moreover, two sections contained adenomas that were classified as tumor. In rare cases, tumor misclassifications occurred, e.g., in areas containing debris and destroyed mucosa tissue (see Fig. 14).

### 4.5 Invasive Margin

**Fig. 15:**
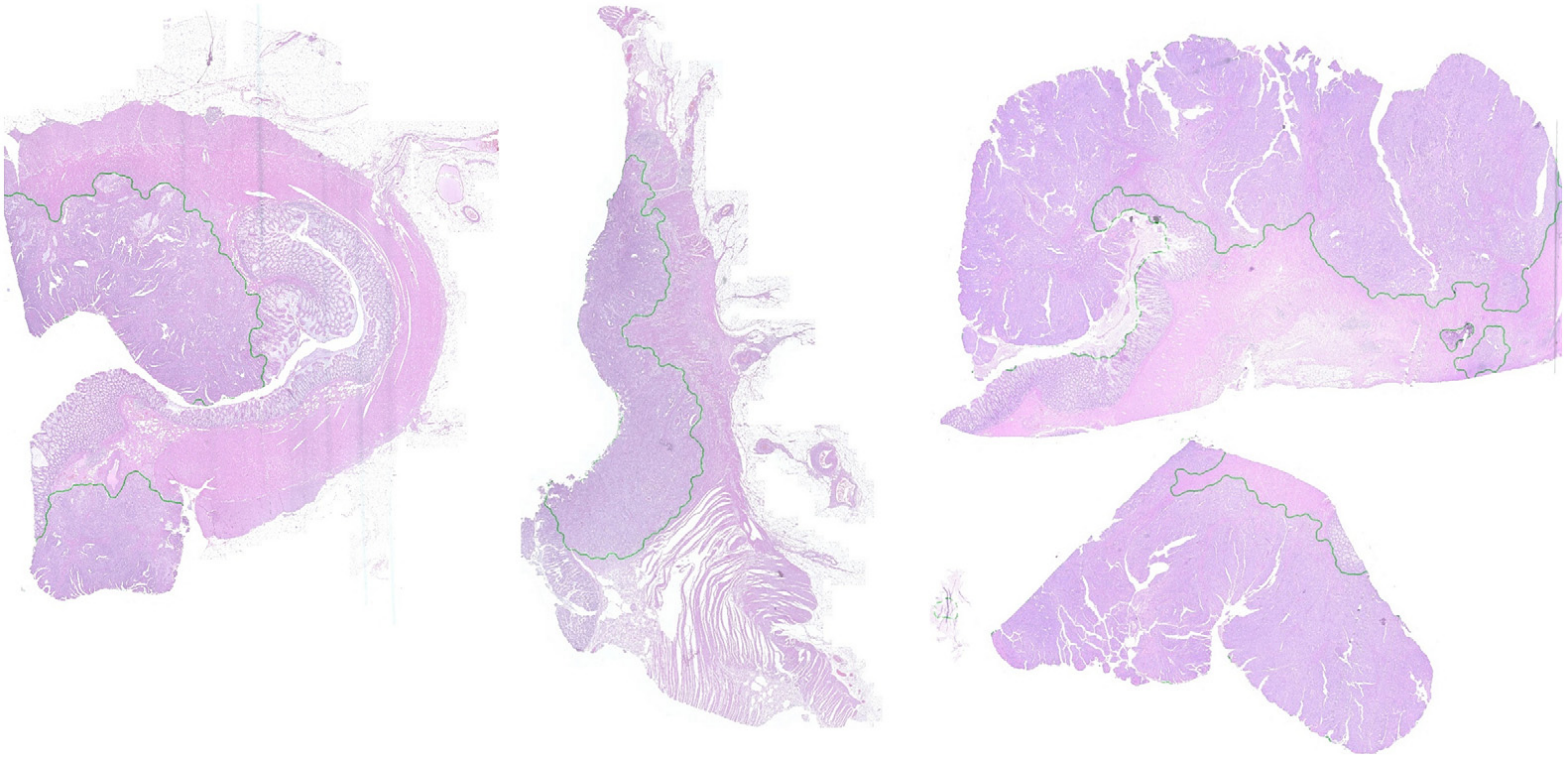
Examples of automatically generated invasive margins (marked in green).

By growing the tumor area evenly by a defined distance toward the surrounding healthy classes, the tumor invasive margin can automatically be generated (see Fig. 15). The generated margins of all slides of dataset B are qualitatively evaluated by two pathologist using a point-based grading system from 1 to 5 (1≙ very good, 5≙ insufficient). On average, the margins were rated 1.6 composed of 18 ratings as “very good,” 15 ratings as “good,” and 3 as “satisfying.” The two pathologists were in correspondence for 13 WSIs and their judgments only differed by one point for five WSIs. These first qualitative results seem promising and could enable further analysis, e.g., the determination of the invasion depth or quantifying inflammation within the invasive margin.

### 4.6 Tumor Composition

**Fig. 16:**
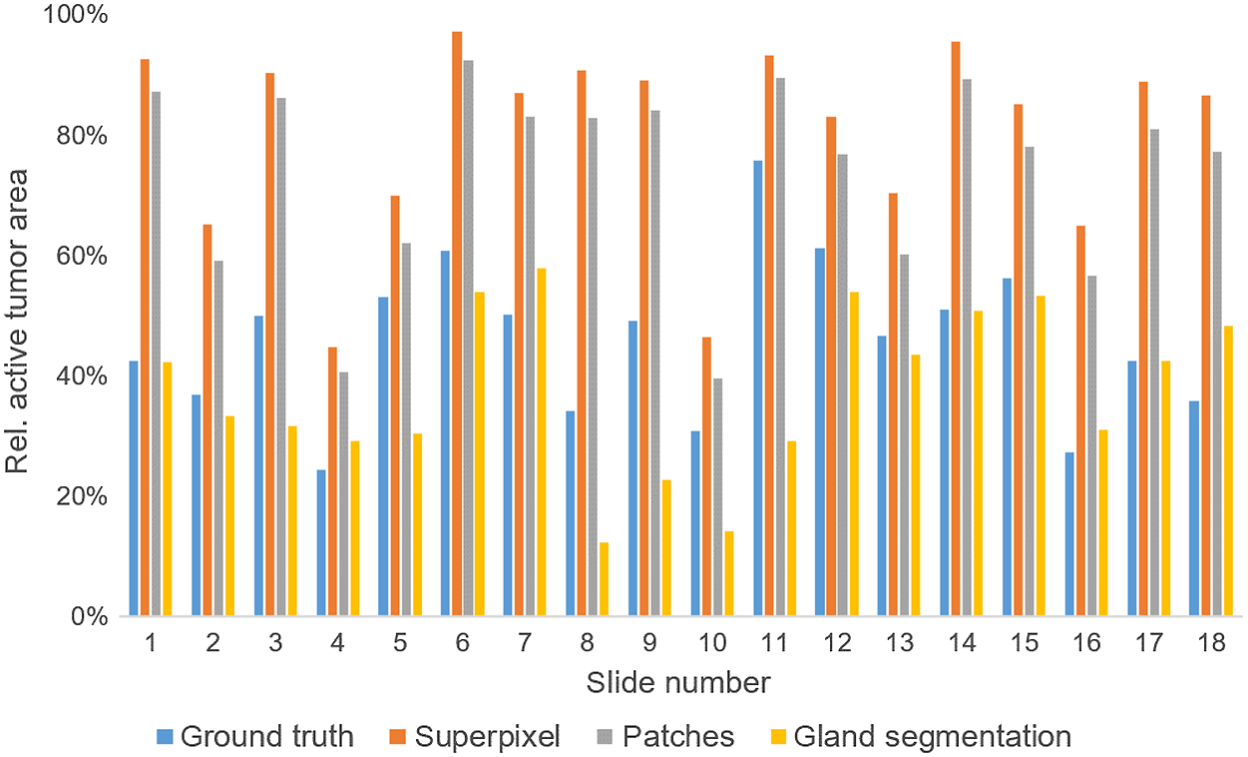
Active tumor area relative to annotated ground truth tumor area calculated with different approaches.

Using dataset B, the tumor composition is evaluated by computing ratios of tumor cells (Fig. 16), necrosis, and mucus within the ground truth tumor area. The results in Fig. 16 show that both the superpixel approach and the patch-based approach overestimate the active tumor area for every slide. However, the average deviation is smaller in the latter case. The best estimation is obtained with the gland segmentation approach.

To analyze these results further, the slides of dataset B have been divided into subsets according to their tumor grading. Table 4 breaks down the deviation of estimated active tumor area from the ground truth for each subset as mean over the subset. For tumors with grade 1 and grade 2, the gland segmentation approach provides good estimations of the active tumor area. As expected, the accuracy decreases with tumor belonging to grade 3 where the growth becomes diffuse and gland structure is destroyed.

**Table 4:** Comparison of different methods for the determination of active tumor area. The average deviation between the calculated relative active tumor area and the ground truth relative active tumor area is given. Slides are assigned to the set “grade 3” as soon as parts with grade 3 are present.

Grade	Superpixel (%)	Patch-based (%)	Gland segmentation (%)
1 to 2	34.7	28.3	3.7
3	33.2	26.9	21.1
Dataset B	34.0	27.7	11.4

**Fig. 17:**
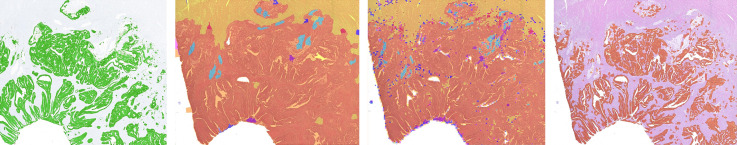
Comparison of active tumor areas obtained with different approaches for slide number 1 with a tumor of grade 2. From left to right: ground truth segmentation (green) of active tumor area in IHC staining. Cartography results in H&E staining by superpixel approach and patch-based approach. Gland segmentation results. Active tumor area is depicted in orange for all three approaches.

**Fig. 18:**
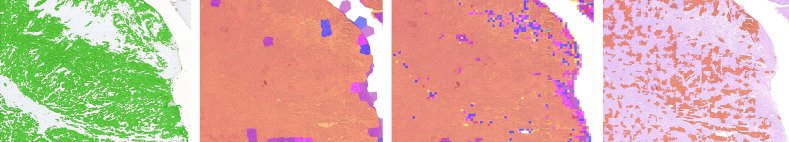
Comparison of active tumor areas obtained with different approaches for slide number 11 with a tumor of grade 3. From left to right: ground truth segmentation (green) of active tumor area in IHC staining. Cartography results in H&E staining by superpixel approach and patch-based approach. Gland segmentation results. Active tumor area is depicted in orange for all three approaches.

Figure 17 shows the detected active tumor area (marked in orange) for a well-differentiated tumor (grade 2, slide number 1) for all three approaches. This example illustrates that the superpixel approach overestimates the active tumor area due to misclassification of tumor stroma as tumor cells. The patch-based approach shows a similar behavior, however, with smaller deviations to the ground truth. The gland detection approach is in good correspondence with the ground truth segmentation. The limitation of this approach is evident in the second example (Fig. 18) showing a tumor with grade 3 (slide number 11). In this example, the estimated active tumor area deviates significantly from the ground truth area (Table 4). On the contrary, the deviation to the ground truth for the superpixel and patch-based approaches seems to be independent of the grade of the tumor.

Besides the active tumor area, the ratio of necrosis and mucus area within the tumor area are additional relevant parameters for characterization of the tumor microenvironment. Both, the patch-based and superpixel approach show similar results here with a slight superiority of the patch-based approach for the determination of the necrotic area (see Table 5). Because the average superpixel size ($0.048\;{\mathrm{mm}}^{2}$ on dataset B) is significantly bigger than the patch size ($0.002\;{\mathrm{mm}}^{2}$) necrotic areas, which are oftentimes only small islands between tumor cells, seem to be better captured by the patches than the superpixels.

**Table 5:** Comparison of superpixel and patch-based approach for the determination of necrotic and mucus area. Necrotic areas were present in all of the slides. However, only five slides contained mucus areas. Therefore, the average deviation for the relative mucus area is calculated once for all slides and once only for slides containing mucus.

Average deviation	Superpixel (%)	Patch-based (%)
Rel. necrotic area	1.77	1.22
Rel. mucus area (all WSIs)	0.19	0.24
Rel. mucus area (only WSIs containing mucus)	0.56	0.55

## 5 Conclusion

In this work, we presented an approach for histology whole-slide cartography using superpixels by the example of colon carcinomas. Our work was motivated by a feasibility of the developed method in a clinical setting. Even though, regarding granularity of segmentation outputs, encoder–decoder-based approaches are sometimes considered superior to patch-based approaches, they often require powerful hardware not attainable in a clinical setting. Therefore, our work focused on increasing the efficiency of a patch-based cartography, which can easily be transferred to, e.g., a pathology institute and ensures fast inference. This increased efficiency could be obtained by presegmenting the input image into superpixels and only classifying a subset of patches within these superpixels.

The evaluation results on our test set composed of 29 WSIs show a superiority of our approach compared to a classical patch-based approach for overall accuracy with an increase from 93.8% to 95.7% as well as computing-time with an average speed-up of 41% resulting in an average overall run-time per WSI of 7.5 min. The speed-up is mainly achieved by limiting the number of classified patches within each superpixel. This patch restriction only results in a marginal decrease in accuracy of 0.3% points compared with the unrestricted approach. These results indicate that the superpixel clustering already segments the WSI into regions belonging to the same tissue type. Only when this requirement is fulfilled can accurate cartography results be obtained. The limitation of our approach lies in the relatively large size of superpixels compared to patches. On our test set one superpixel on average covers $0.005\;{\mathrm{mm}}^{2}$. Compared to fine-grained structures, such as small necrotic areas within the tumor, this size is too big to correctly capture these areas. This is also reflected in, e.g., a lower recall for necrosis. Another limitation lies in the manual annotations. Accurate and complete annotation of these fine-grained structures are also a challenge for the human annotator. Therefore, wherever possible, an alternative generation of the ground truth should be preferred, e.g., based on segmentation in immunohistochemically stained sections. Moreover, one has to keep in mind that there is a general problem with the quantitative assessment of cartography results. Although using seven tissue classes, there are still areas that cannot be clearly assigned to one of these classes. These nonannotated areas are not included in the quantitative evaluation. Therefore, from our point of view, it is important to always apply the developed approaches to complete WSIs and check the cartography results in these areas at least qualitatively.

The key difference of our method compared with other superpixel-based approaches for histopathology images is the one-to-many relationship between superpixels and corresponding image patches. In our setup, a superpixel contains on average 20 image patches of which we classify a random subset. Utilizing the fact that a superpixel class-label is inferred from a set of multiple individually classified patches, we investigated a measure for quantifying the uncertainty of a superpixel classification derived from the votes of the patches within the superpixel. This measure was suited to decrease the relative number of incorrect predictions at the cost of introducing unclassified tissue areas and a rejection of correct predictions, but to a smaller extent. Moreover, applying our introduced uncertainty measures to WSIs and visualizing uncertain superpixels enables a plausibility check of the approach. As expected, the classification results of superpixels in the transition of two tissue types, e.g., located near the invasive margin, tend to be unsure. The uncertainty measurement also facilitates an automatic improvement by, e.g., partitioning them into smaller segments and reclassifying these superpixels or applying other pixelwise segmentation methods within these areas.

Whole-slide cartography by itself offers only limited support to the pathologist but provides a basis for subsequent analysis operations that can predict various medical endpoints. Within this work, we used the cartography results to determine the tumor area and composition as well as to derive the invasive margin. While the tumor area is in good agreement with the ground truth, the tumor composition analysis highlights weaknesses of the approach. Again, due to the size of our superpixels, the separation between finely grained active tumor cells and tumor stroma is not adequate. However, a combination with further methods (in our case gland segmentation for well differentiated tumors) yields good results with an average deviation of only 11.4%.

Being able to reliably detect and outline, the tumor area is very valuable from a clinical perspective. In a routine workflow, such a functionality could be used as an assistance system that draws the pathologist’s attention to a specific region. Alternatively, such a system could be introduced as a quality control mechanism that provides a second opinion. Another potential application is the insurance that samples for molecular testing are taken from an area that actually contains a high ratio of tumor cells. In the context of computational pathology, it has been shown in recent literature that it is possible to predict genetic alterations directly from WSIs.^50^^,^^51^ A prerequirement here is that only tumor-areas are analyzed. In the context of colon carcinoma, an example would be the detection of microsatellite instability to validate the presence of Lynch syndrome. The tumor composition in terms of the ratios of active tumor cells, tumor stroma, necrosis, and mucus has been shown to be of prognostic relevance^52^ and could at least for well-differentiated tumors be assisted by the proposed approach.
